# Advances in the Production of D-Tagatose Using L-Arabinose Isomerase

**DOI:** 10.3390/microorganisms14092085

**Published:** 2026-09-17

**Authors:** Bingyi Tao, Wei Liu, Ziheng Li, Ren He, Tingting Huang, Shaoxiong Liang, Hongkun Chen, Xiaoping Rao, Xuchong Tang, Jianchun Jiang

**Affiliations:** 1 College of Chemical Engineering, Huaqiao University, Xiamen 361021, China; 2 Fujian Provincial Key Laboratory of Biomass Low-Carbon Conversion, Academy of Advanced Carbon Conversion Technology, Huaqiao University, Xiamen 361021, China; 3 BaYeCao Health Industry Research Institute Co., Ltd., Xiamen 361021, China; 4 Institute of Chemical Industry of Forest Products, Chinese Academy of Forestry, Nanjing 210042, China

**Keywords:** D-tagatose, L-arabinose isomerase, biocatalysis

## Abstract

D-tagatose is a rare sugar that occurs naturally and combines high sweetness with a low caloric value. It has several beneficial effects, including anticariogenic activity, modulation of the intestinal microbiota, and potential benefits for diabetes management. It is used as a novel sweetener and pharmaceutical excipient in the food and pharmaceutical industries. Currently, D-tagatose is produced mainly through chemical synthesis and biocatalysis. The chemical preparation of D-tagatose requires complex product purification and entails high waste-treatment costs, while biological preparation of D-tagatose offers high catalytic efficiency, mild reaction conditions, and fewer by-products. Therefore, in recent years, the biocatalytic conversion of D-galactose into D-tagatose using L-arabinose isomerase (L-AI) has attracted considerable attention, prompting extensive efforts to develop more economical and environmentally friendly production methods. To date, L-AI from various microbial sources has been identified and characterized, and researchers have engineered L-AI into a more effective biocatalyst through mutagenesis to improve process feasibility. This paper comprehensively reviews the current status and the latest advancements in the synthesis of D-tagatose from the perspective of biocatalysis.

## 1. Introduction

The incidence of diabetes and obesity has increased dramatically worldwide over the past few decades, increasing the demand for low- or zero-calorie sweeteners among people with diabetes or overweight. It is urgent to research and develop new low-calorie functional sweeteners. Rare sugars are defined as a group of monosaccharides and their derivatives that exist in nature but in very small quantities [1]. Their sweetness is similar to that of sucrose, and they have the advantages of low calorie content, high stability, well-balanced sweetness, non-hygroscopicity, non-cariogenicity, and high tolerance. They can make up for the deficiencies of traditional sweeteners, and play an important role in the fields of diet, medicine, and health care [2]. At present, about 50 types of rare sugars have been discovered, and the more commonly studied ones are D-psicose, D-tagatose, trehalose, xylitol and L-sorbose [3].

D-tagatose is a rare natural hexose, the C-4 epimer of D-fructose, and an aldo-keto isomer of D-galactose. Pure D-tagatose is a white anhydrous crystalline substance with a melting point of 133–137 °C. It is odorless, soluble in water, and slightly soluble in ethanol. In a 10% aqueous solution, the sweetness of D-tagatose is similar to that of sucrose, providing approximately 92% of its sweetness without an unpleasant aftertaste, while contributing only about 30% of its caloric value [4]. D-tagatose is stable at pH > 3.0. In addition, as a reducing sugar, D-tagatose is prone to the Maillard reaction and caramelization at elevated temperatures [5]. In 2001, the U.S. Food and Drug Administration recognized D-tagatose as generally recognized as safe (GRAS) [6]. It has been reported to be noncarcinogenic, to promote the growth of lactic acid bacteria in the intestinal tract of humans and animals, and to exhibit antidiabetic effects and potential benefits in preventing lifestyle related diseases [7]. Therefore, D-tagatose is widely used in food and pharmaceutical applications. The production of D-tagatose includes both chemical and biological methods. Chemical methods have been used commercially, but disadvantages include complex purification, by-product formation, and high waste disposal costs [8]. Biological routes using microorganisms and enzymes have become a major research focus, and enzyme engineering has been used to improve catalytic performance. Therefore, this paper systematically and comprehensively reviews the current status and progress of research on the synthesis of D-tagatose in recent years from the perspective of biocatalysis. The research progress on the synthesis of D-tagatose by L-AI from different microbial sources is comprehensively discussed, and feasible strategies for increasing the yield of D-tagatose and reducing production costs are systematically sorted out, providing ideas and directions for the research on the sustainable production of D-tagatose.

## 2. D-Tagatose

### 2.1. Properties of D-Tagatose

**Figure 1:**
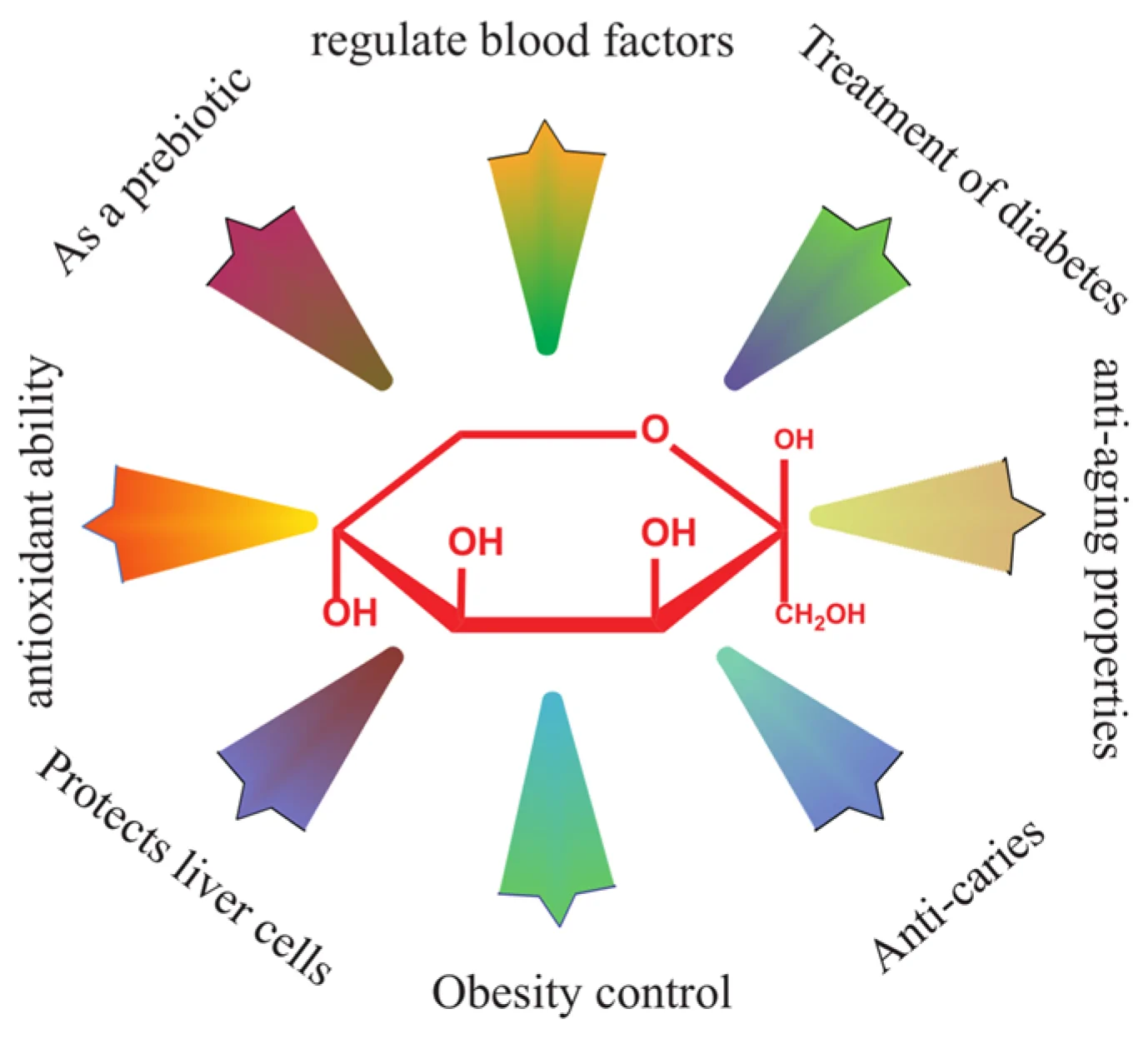
Physiological functions of D-tagatose.

D-tagatose provides several physiological benefits, including disease management (diabetes treatment and obesity control) and health promoting effects (anticariogenic activity and prebiotic effects) (Figure 1) [9]. D-tagatose is suitable for individuals with diabetes and can be used as a sweetener and nutritional additive in diabetic foods. Studies have shown that in diabetic patients, the intake of D-tagatose does not markedly increase insulin levels and can effectively inhibit the absorption of glucose and other substances in the intestine, thus controlling the blood glucose level [10]. Therefore, D-tagatose may support diabetes management. Prebiotics are nondigestible dietary components that benefit the host by selectively stimulating beneficial members of the gut microbiota [11]. Only about 20% of ingested D-tagatose is absorbed, whereas the remaining 80% reaches the large intestine and is subsequently metabolized by beneficial gut microorganisms (such as lactic acid bacteria), thus lowering the pH of the intestine and increasing the concentration of ATP in the intestine, thereby promoting beneficial microorganisms and inhibiting pathogenic bacteria such as *Escherichia coli,* and supporting intestinal digestion and nutrient absorption [12]. As a low-calorie sweetener, D-tagatose may help prevent the development of obesity. Experimental studies have found that most of the tagatose entering the human body is fermented in the large intestine to produce short chain fatty acids, which can then re-enter the body’s metabolic processes [3]. In 2003, PepsiCo used D-tagatose as a flavor additive in low energy beverages to reduce caloric content while retaining the original flavor. D-tagatose is poorly metabolized by microorganisms in the oral cavity. Some scholars found that adding D-tagatose to toothpaste can effectively disrupt oral microbial biofilms, and the pH of dental plaque remains relatively stable after use, thereby limiting enamel demineralization and reducing the occurrence of oral diseases such as dental caries [13]. In addition, D-tagatose can also regulate blood-related factors, thereby supporting vascular health and effectively preventing cardiovascular and cerebrovascular diseases [8].

### 2.2. Metabolic Pathway of D-Tagatose

Although the human intestine contains a diverse microbiota, only a small proportion of microorganisms can ferment D-tagatose [14], such as *Enterococcus faecalis*, *Enterococcus faecium* and *Lactobacillus*. Many lactic acid bacteria found in dairy products can ferment D-tagatose, such as *Pediococcus* and *Lactobacillus*, which generally show strong metabolic capacity, whereas *Enterococcus* and *Lactococcus* generally show weaker metabolic capacity.

**Figure 2:**
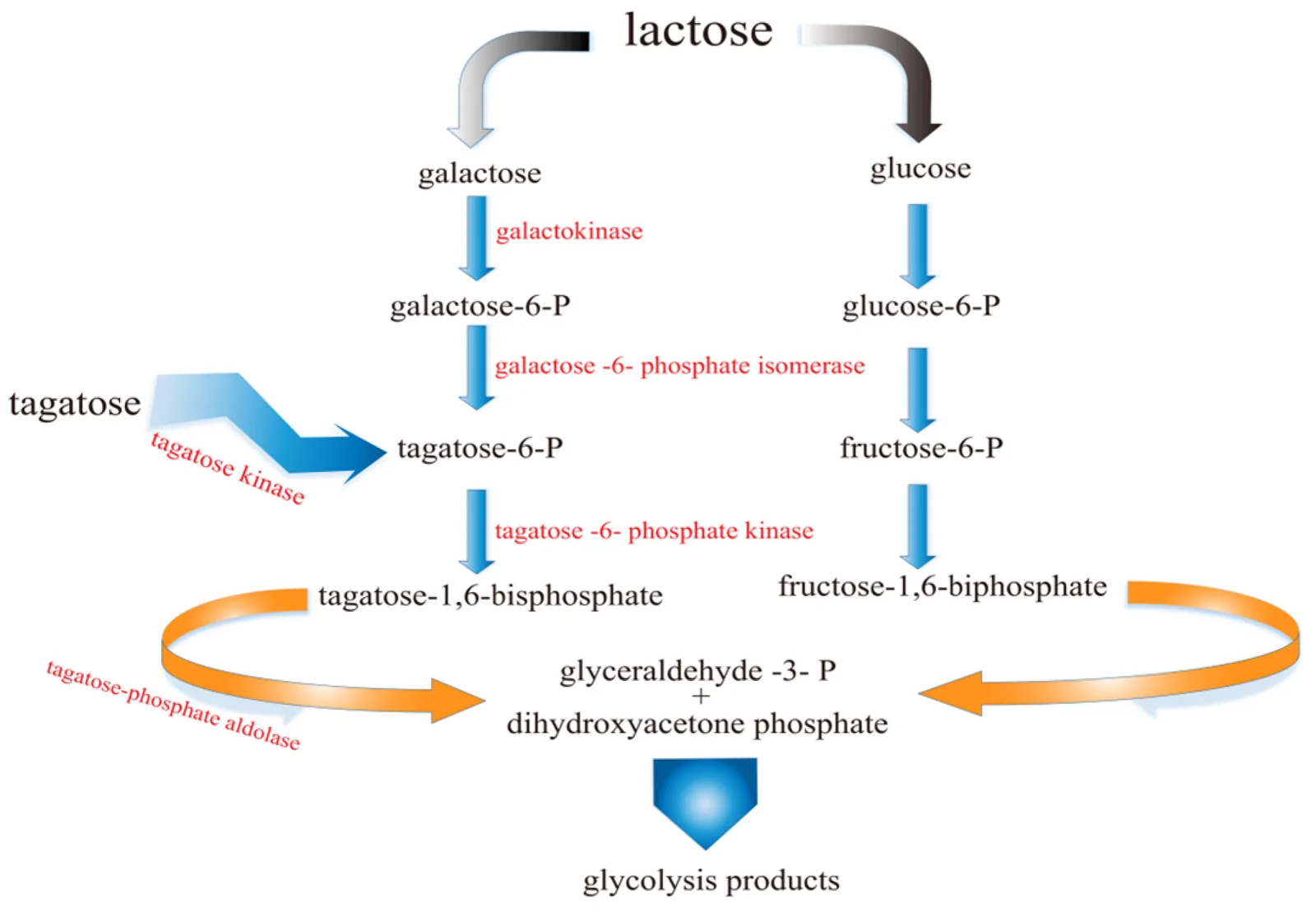
Tagatose-6-phosphate pathway.

In microorganisms, D-tagatose is fermentatively metabolized via the tagatose-6-phosphate pathway (Figure 2), which is an offshoot of the Leloir pathway that catabolizes lactose to galactose [15]. In the tagatose-6-phosphate pathway, lactose is broken down into glucose and galactose. After that, galactose generates tagatose-6-phosphate through the action of galactose kinase and galactose-6-phosphate isomerase. In contrast, absorbed D-tagatose generates tagatose-6-phosphate via tagatose kinase, which then enters the tagatose-6-phosphate pathway. Then, tagatose-6-phosphate is converted to tagatose-1,6-bisphosphate by tagatose-6-phosphate kinase. It then passes through tagatose-phosphate aldolase, generating dihydroxyacetone phosphate and glyceraldehyde-3-phosphate, and finally enters the glycolysis pathway [16].

**Figure 3:**
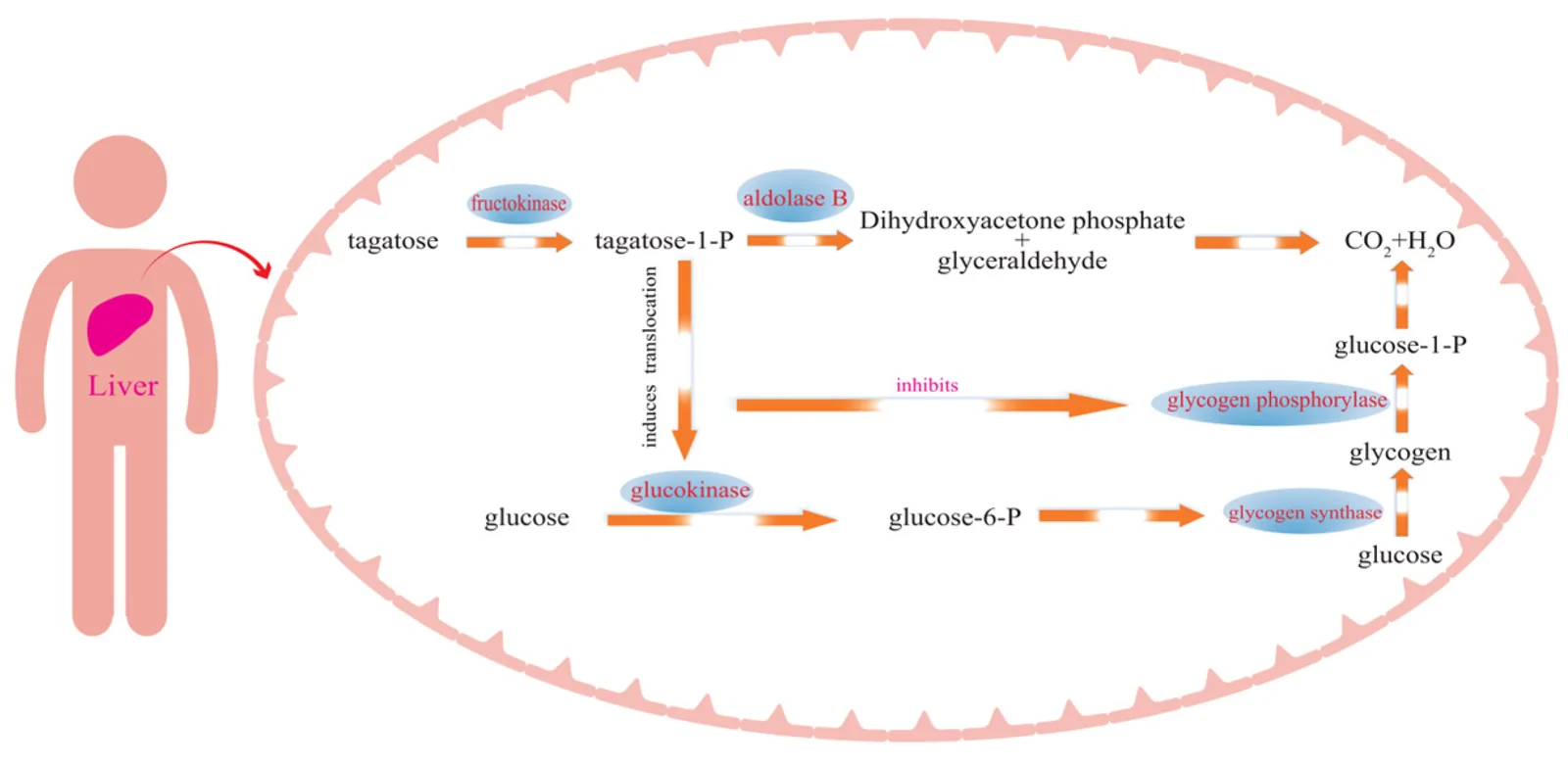
D-Tagatose metabolic pathway.

In higher animals, including humans, a small amount of tagatose absorbed by the small intestine is mainly metabolized in the liver (Figure 3) [17]. D-tagatose is phosphorylated by fructokinase and generates tagatose-1-phosphate, and then glyceraldehyde and dihydroxyacetone phosphate are produced by aldolase B. This metabolic process is similar to that of fructose, but because aldolase B has a stronger affinity for fructose-1-phosphate, the catabolism efficiency of fructose will be somewhat higher than that of tagatose. Through the study, it was found that tagatose-1-phosphate can induce the transfer of glucokinase from the nucleus to the cytoplasm, catalyze the phosphorylation of glucose to generate glucose-6-phosphate and activate glycogen synthase [18]. In addition, tagatose 1-phosphate inhibits the activity of glycogen phosphorylase, thereby reducing glycogen utilization.

### 2.3. Production Methods for D-Tagatose

**Figure 4:**
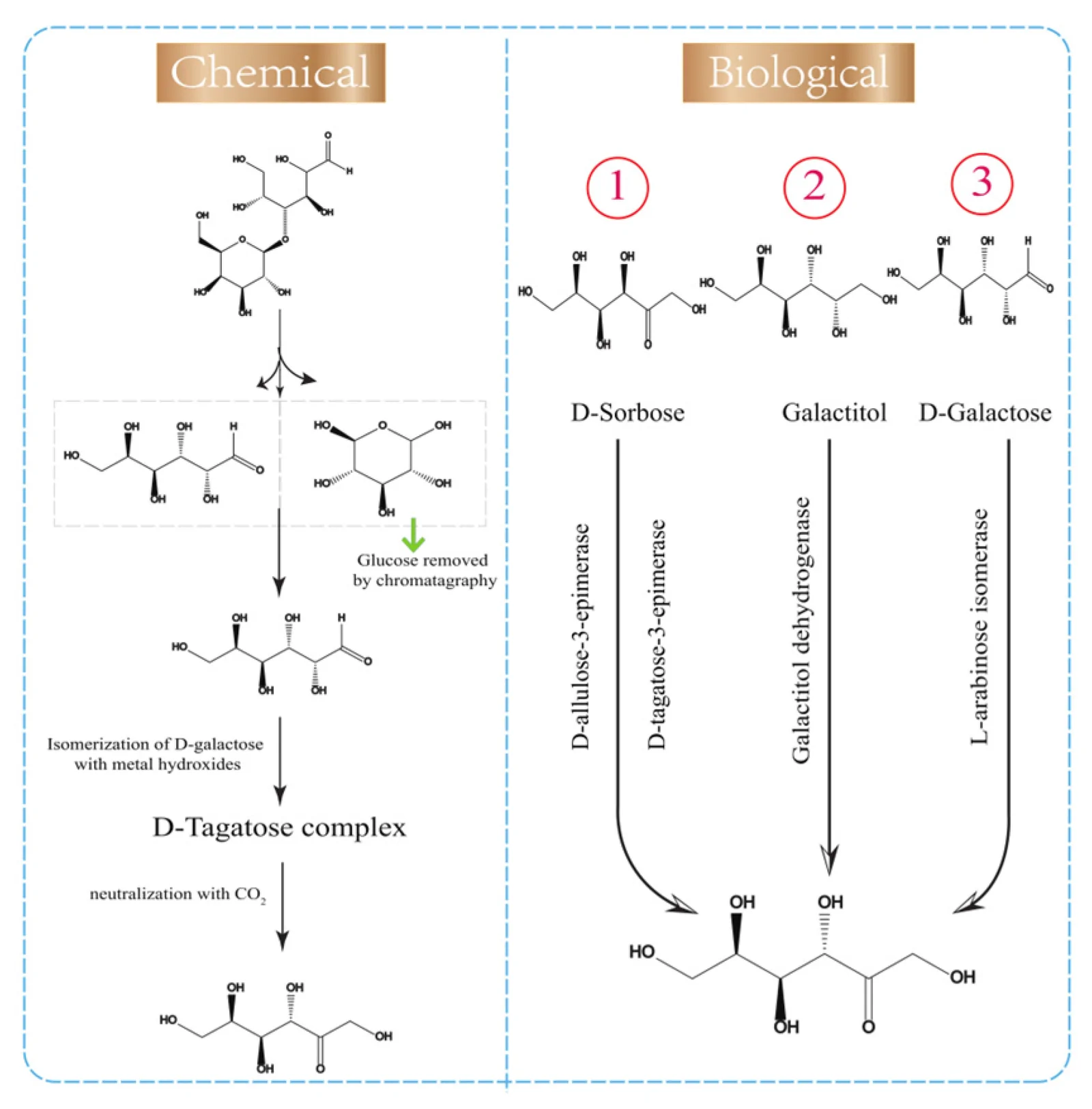
Roadmap for the synthesis of tagatose by chemical and biological methods.

D-tagatose occurs only in small amounts in nature. It was first identified in gum secreted by *Sterculia setigera* and was later detected in lower organisms such as lichens and mosses and in dairy products such as milk and yogurt. Therefore, extraction from natural sources cannot meet industrial demand. Chemical synthesis of D-tagatose was first proposed in the late 19th century [19]. In the chemical process of tagatose production, galactose is used as the raw material in a two-step process (Figure 4). The first step is the isomerization of D-galactose, in which D-galactose is reacted with a metal hydroxide (Ca(OH)_2_ or a mixture of Ca(OH)_2_ and NaOH) in the presence of a catalyst (CaCl_2_ or NaCl) to form an insoluble D-tagatose complex, which is stable in alkaline media. The second step is acid neutralization, in which the reaction mixture is acidified to release D-tagatose and form insoluble salts. D-tagatose is then separated from the salts by filtration [20]. However, the chemical process for the production of D-tagatose has drawbacks associated with product purification, chemical waste treatment, and by-product formation [21]. These limitations have motivated the development of biological methods for D-tagatose production.

Biological methods can effectively overcome these shortcomings, while also offering the advantages of high specificity, high catalytic efficiency, and mild reaction conditions [22]. Currently, the biological production of D-tagatose can be classified into three categories based on raw materials: the first method, proposed by Izumori et al. [23] in 1984, uses galactitol as the raw material and oxidizes it into D-tagatose via galactitol dehydrogenase. The second method was developed by Kim et al. [24], who synthesized D-tagatose using D-sorbose as the raw material in the presence of D-allulose-3-epimerase and D-tagatose-3-epimerase. However, both substrates are expensive and not suitable for the industrial production of tagatose. The third method, put forward by Cheetham et al. [25], uses D-galactose as the raw material and isomerizes it to D-tagatose via L-arabinose isomerase. Since galactose can be obtained by decomposing low-cost whey, it is more suitable as a raw material for the industrial production of D-tagatose [26]. At present, L-AI is the most effective enzyme for producing D-tagatose through biotransformation. The activity of L-AI can be enhanced by screening high-yield L-AI-producing strains, constructing genetically engineered bacteria, and carrying out directed molecular modification of the enzyme, so as to improve the conversion rate and yield of tagatose and make the process suitable for industrial production.

## 3. L-Arabinose Isomerase

### 3.1. Properties of L-AI

**Figure 5:**
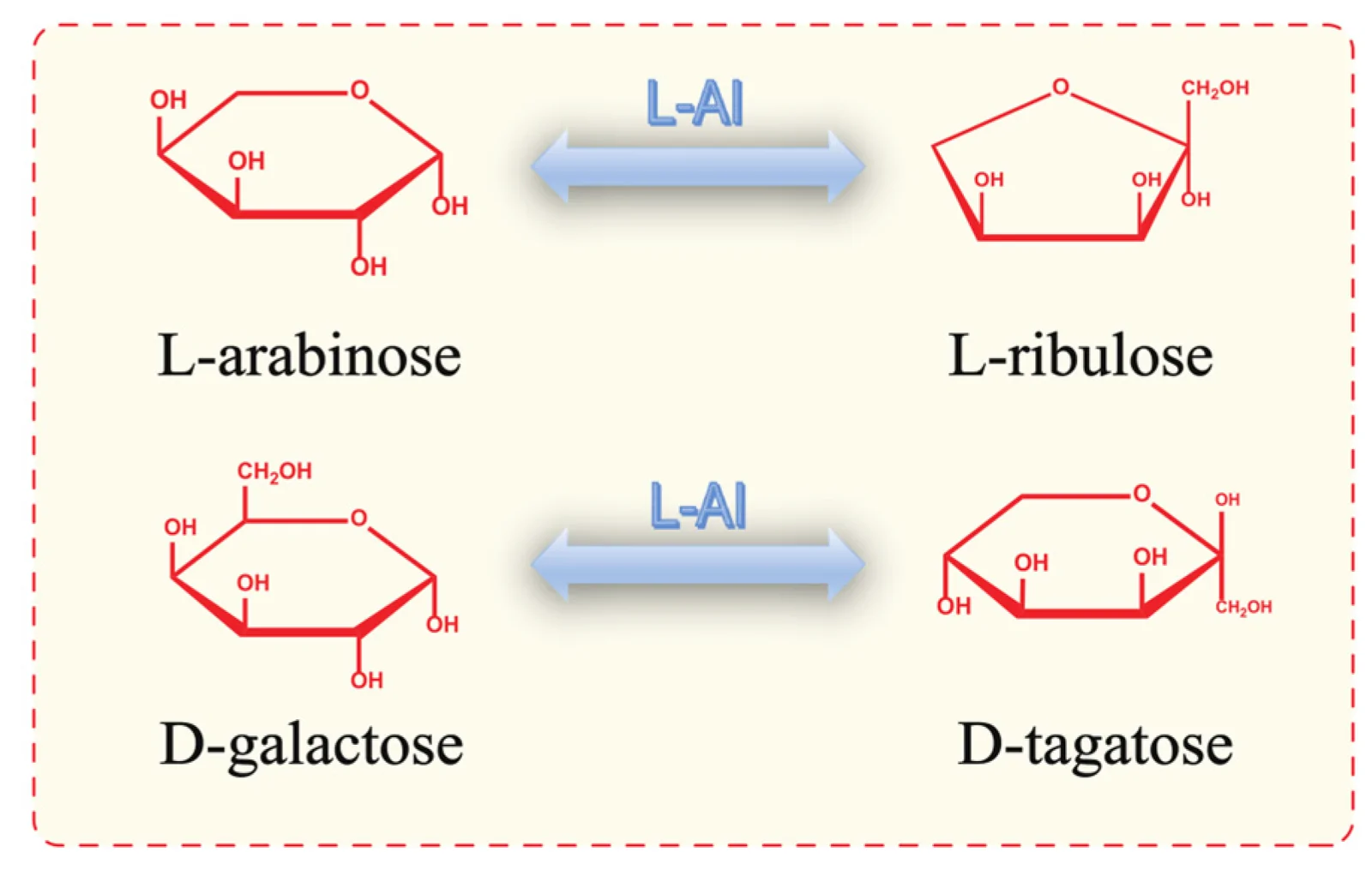
L-AI-catalyzed isomerization reaction.

L-arabinose isomerase (EC 5.3.1.4, L-AI) is widely distributed among microorganisms and is an intracellular enzyme that catalyzes the isomerization of L-arabinose to L-ribulose. Since D-galactose and L-arabinose are structurally similar, L-AI can also convert D-galactose to D-tagatose (Figure 5), but overall, L-AI generally has a higher affinity for L-arabinose than for D-galactose [27].

**Figure 6:**
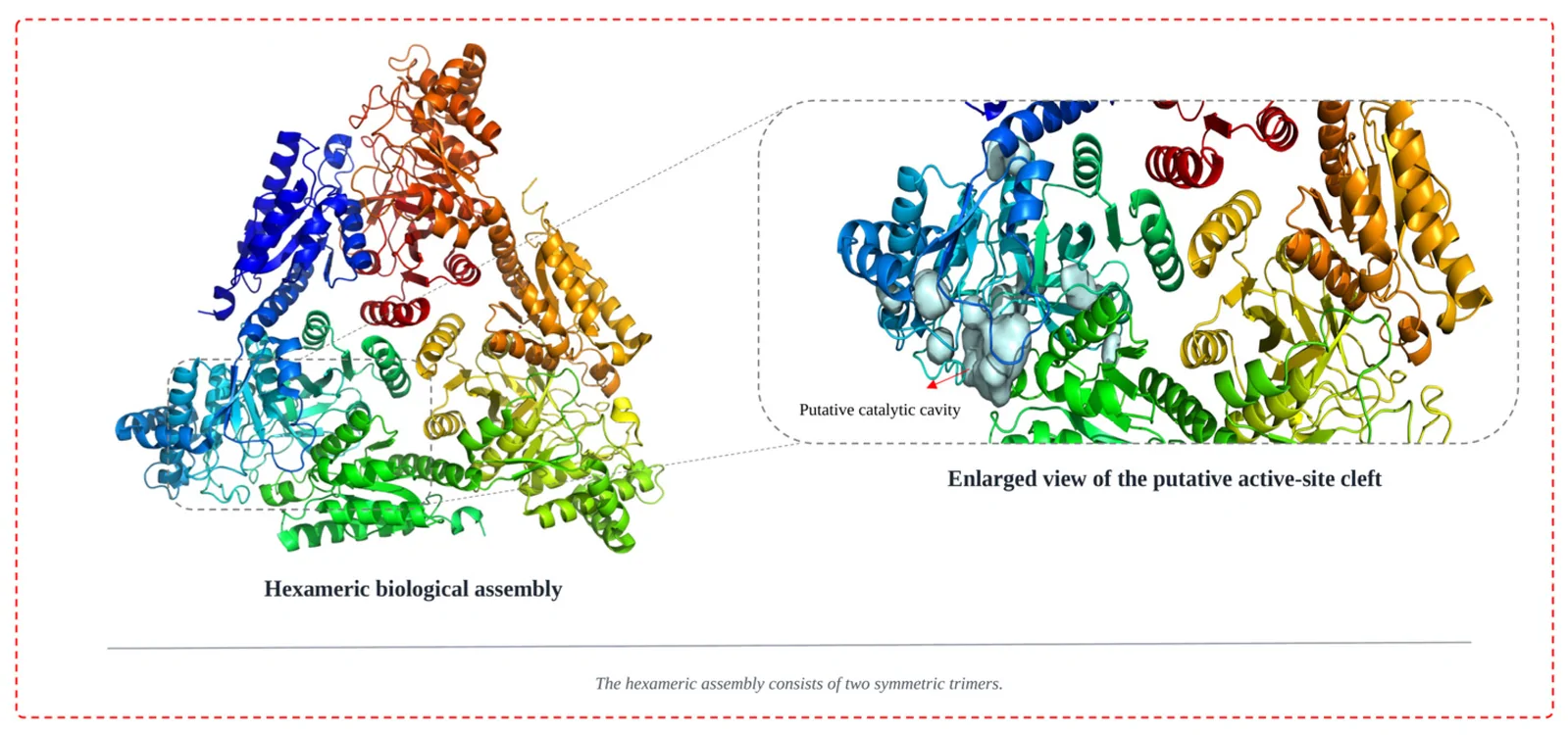
Three-dimensional structure of *E. coli* L-arabinose isomerase. The structural coordinates were downloaded from the RCSB Protein Data Bank (PDB ID: 2AJT) and visualized by the authors using PyMOL 3.1.6.1. The crystal structure was originally determined by Manjasetty and Chance [29].

In 1969, Patrick et al. [28] first reported the structure of L-AI from *E. coli*, and they concluded that each L-AI molecule comprises six identical subunits with a molecular weight of 362 kDa. In 2006, Manjasetty et al. [29] further determined the crystal structure of L-AI from *E. coli* (PDB ID: 2AJT), and they found that L-AI consists of three domains: an N-terminal domain (residues 1–176), an intermediate domain (residues 177–327), and a C-terminal domain (residues 328–500), with a total of 16 β-folds and 17 α-helices. They also found that the six L-AI subunits form two symmetrical trimers, which assemble into a symmetric prism-like structure with three equivalent clefts on its outer surface and clefts within the subunits located between the three structural domains, thereby forming a pronounced electron density cavity that is presumed to contain the enzyme’s active center (Figure 6).

### 3.2. Sources of L-AI

L-AI is present in most prokaryotic microorganisms, including *E. coli*, *Lactobacillus* (*Lactobacillus plantarum*, *Lactobacillus fermentum*, *Lactobacillus sakei*, etc.), *Bifidobacterium longum*, thermophilic bacteria (*Geobacillus stearothermophilus*), hyperthermophilic bacteria (*Thermotoga maritima*, *Thermotoga neapolitana*, etc.), and *Bacillus* (*Bacillus subtilis*, *Bacillus stearothermophilus*, etc.). Table 1 summarizes the properties and optimum reaction conditions of L-AIs from different microbial sources. Recent genome-mining efforts have continued to expand the diversity of L-AIs available for D-tagatose synthesis. Nirwantono et al. [30] characterized an L-AI from the cryophilic bacterium *Arthrobacter psychrolactophilus* B7. The recombinant enzyme exhibited high thermal stability and an unusual homopentameric organization, highlighting additional structural diversity among L-AIs. Table 1 shows optimum temperatures spanning 30–95 °C. L-AIs with optimal around 30–50 °C can be described as mesophilic, those around 60–80 °C as thermophilic, and those at 85–95 °C as the hyperthermophilic group. Thus, *L. sakei* 23K (30–40 °C) is mesophilic; *L. fermentum* CGMCC2921 (65 °C), *B. coagulans* (70 °C), and *B. stearothermophilus* US100 (80 °C) are thermophilic; and *T. neapolitana* (85 °C), *T. maritima* (90 °C), and *A. flavithermus* (95 °C) fall in the highest-temperature group. In addition, most enzymatic reaction processes require the participation of metal ions, and the more common metal ion activators are Mn^2+^, Co^2+^, etc.

### 3.3. L-AI and D-Tagatose Production

As shown in Table 2, the conversion rate of L-AI from *L. brevis* PC16 reached 79.7% after 28 h of reaction, and the D-tagatose titer reached 99.6 g/L [4], reflecting the enzyme’s substrate adaptability and reaction stability toward D-galactose. L-AI from *B. subtilis* also demonstrated excellent product accumulation ability, achieving a conversion rate of 57.2% and a D-tagatose titer of 96.8 g/L after 40 h of reaction [44]. Notably, *L. plantarum* expressing *L. sakei*-derived L-AI achieved a conversion rate of 85% after 48 h, but the D-tagatose titer was only 45.9 g/L [45]. The high conversion rate but low product titer may be due to differences in substrate loading in the reaction system. The L-AI conversion rates from *L. plantarum* WU14, *B. coagulans* NL01, and *L. parabuchneri* CICC 6004 ranged between 32% and 40.28%, at a temperature of 45–60 °C [32,40,43]. In contrast, the conversion rate of L-AI from *A. flavithermus* reached 60% after 1 h of reaction at 95 °C [35]. This is due to the enzyme’s excellent inherent thermal stability and high catalytic efficiency, indicating that as the reaction temperature increases, the isomerization of D-galactose to D-tagatose catalyzed by L-AI becomes more favorable for D-tagatose production. Overall, the application potential of L-AI from different microbial sources in D-tagatose synthesis varies significantly. Enzyme performance should be evaluated through multiple parameters, including conversion rate, D-tagatose titer, productivity, substrate loading, catalytic efficiency, operating conditions, and stability or reusability.

Although the optimum reaction pH of L-AI has been reported to be in the range of 5.0–10.5 and generally falls within 7.0–8.0, acidic catalytic conditions are preferred to meet industrial production requirements. This is because lactose or whey is usually used as raw material in the production of D-tagatose, where lactose is first hydrolyzed to galactose and glucose, and then L-AI is used to isomerize D-galactose to D-tagatose [46]. The hydrolysis of lactose in the first reaction step is usually carried out under acidic conditions at pH 5–6. The reaction of L-AI under acidic conditions eliminates the need for pH adjustment before the second reaction step [27]. In addition, acidic conditions can reduce the occurrence of side reactions, including the Maillard reaction, so identifying L-AIs that remain active in acidic environments to produce D-tagatose can reduce production costs. D-tagatose itself is commercially available, but published L-AI-based production remains dominated by laboratory and process-development studies, and broad industrial implementation of L-AI processes is still limited [20]. In the future, it is necessary to start from the molecular level and utilize genetic engineering, protein engineering, and other related technologies to modify the molecular structure of L-AI, altering conditions such as its optimal reaction temperature and pH value, so as to achieve large-scale production of D-tagatose.

**Table 2:** Production of D-tagatose by L-AI from different microbial sources.

Microorganism Source	Optimal Temperature (°C)	Optimal pH	Reaction Time (h)	Conversion Rate (%)	D-Tagatose Titer (g/L)	References
*L. brevis* PC16	67	6.5	28	79.7	99.6 *	[4]
L. *parabuchneri* CICC 6004	45	6.7	100	39	54.0	[40]
*E. coli* expressing L-AI from *K. pneumoniae*	50	8.0	0.5	33.5	33.5	[21]
*L. plantarum* WU14	60	7.17	24	40.28	58.0	[32]
*A. flavithermus*	95	10.5	1	60	-	[35]
*B. subtilis*	50	7.0	40	57.2	96.8	[44]
*E. coli* expressing L-AI from *L. parabuchneri*	45	6.7	50	39	39.0	[40]
*S. flexneri*	40	8.0	24	22.3	-	[41]
*B. coagulans* NL01	60	7.5	32	32	48.1	[43]
*E. faecium*	50	5.6	96	26	18.9 *	[47]
*L. plantarum* expressing L-AI from *L. sakei*	50	7.4	48	85	45.9 *	[45]
*L. brevis*	65	7.0	23	43	-	[48]
*B. adolescentis*	55	6.5	10	56.7	10.2 *	[49]
*E. faecium* DBFIQ E36	50	4.5–6.0	48	45	40.5 *	[50]

* Approximate D-tagatose titers were calculated only when the cited study explicitly reported initial D-galactose concentration and conversion rate, or a D-tagatose concentration in mM. For molar data, MW = 180.16 g/mol was used; a 1:1 molar isomerization and negligible reaction-volume change were assumed. “-”, Not mentioned.

## 4. Progress Toward Industrial D-Tagatose Production

### 4.1. Engineering of L-AI

**Figure 7:**
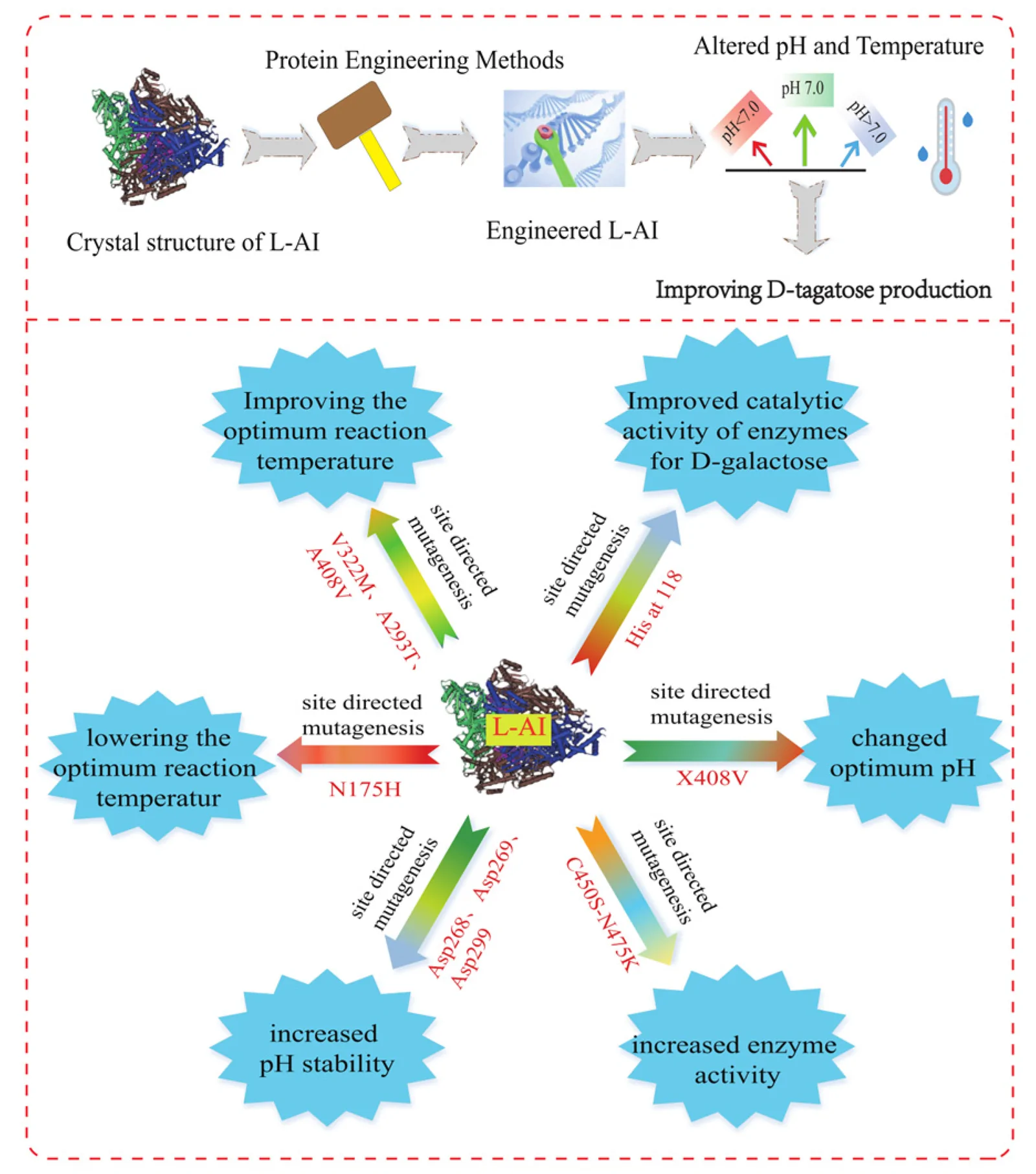
Protein engineering of L-arabinose isomerase from different sources.

Although the characterization of L-AIs from different microorganisms has advanced rapidly in recent decades, their limited stability and catalytic activity at elevated temperatures have constrained their industrial application. Therefore, instead of mining new L-AIs from natural resources, engineering existing enzymes may be a more efficient strategy to meet industrial requirements [20]. Molecular evolution and protein engineering strategies can alter the physicochemical properties of L-AI and can thereby improve the conversion of D-galactose to D-tagatose. Directed evolution is a powerful tool that has been widely used to improve the functional properties of industrial enzymes (Figure 7). Kim et al. [51] mutated the L-AI from *G. stearothermophilus* using error-prone PCR. During the experiment, the V322M, A293T, and A408V substitutions were introduced, and the results showed that the resulting variants displayed improved catalytic parameters, including a substantially higher optimum reaction temperature and increased D-tagatose yield. Because screening large mutant libraries is time consuming and labor intensive and can be affected by experimental variability, researchers have found a suitable alternative to address these problems: structure guided selection of mutation sites near the active site or substrate binding region. From this perspective, Laksmi et al. [52] performed targeted mutagenesis of L-AI from *G. stearothermophilus* to improve the catalytic activity of the enzyme towards D-galactose. His118 was substituted with Thr and it was found that the mutated L-AI had a higher affinity for D-galactose, with 1.8-fold higher activity and 2.3-fold greater catalytic efficiency than the wild type.

Enzyme engineering initially focused on aspects such as increasing catalytic activity and altering regioselectivity. However, the scope of this technology has now gradually expanded to improving enzyme stability by adjusting the optimum reaction temperature and pH [53]. Oh et al. [54] concluded from their study that the amino acid at position 408 is a critical residue for galactose isomerization. They mutated this site and obtained two beneficial variants, Q408V and R408V, which shifted the optimum pH from 8.5 to 7.5, and found that the isomerization activity of Q408V and R408V on D-galactose was 60% and 30% higher than that of the wild type, respectively, which effectively improved the conversion efficiency of D-galactose. Rhimi et al. [55] performed site directed mutagenesis at residue 175 (N175H) of *B. stearothermophilus* US100 L-AI and found that its optimum temperature was lowered from 80 °C to 50–65 °C, which is more suitable for industrial production than the wild type enzyme and can effectively reduce browning side reactions. Xu et al. [56] studied the L-AI from *L. fermentum* and examined the variation in its optimum pH. They selected Asp268, Asp269, and Asp299, which are far from the active site, for mutagenesis and obtained the D268K, D269K, and D299K variants that were able to isomerize D-galactose in the presence of borate at pH 5.0, and found that the D-tagatose yield reached 62%. At the same time, the authors found that these mutations further improved the pH stability. In addition, they found that the half-life increased from 30 h to 62 h at pH 6.0. In addition to enhancing catalytic activity under acidic conditions, targeted mutations can improve thermal stability and thereby increase D-tagatose yield. Shin et al. [57] mutated L-AI from *G. thermodenitrificans* and evaluated the C450S-N475K variant at 65 °C. It was found that the wild-type enzyme produced D-tagatose at a conversion rate of 46%, while the C450S-N475K variant increased the conversion to 58%. More recently, structure guided engineering of *L. plantarum* CY6 L-AI generated the F118M/F279I double mutant, whose specific activity toward D-galactose increased by 210.1% relative to the wild-type enzyme; D-tagatose yield and conversion were also markedly improved [58]. It is clear that advances in molecular biology and protein engineering techniques have had a significant impact on the development of L-AI catalysts for D-tagatose synthesis. Nevertheless, further technological innovation is still required. Currently, computational simulations have become indispensable tools for protein engineers seeking to understand the effects of specific amino acid substitutions [59]. Therefore, solving more L-AI crystal structures and developing new simulation tools will be essential for advancing protein engineering and improving D-tagatose yields.

### 4.2. Use of Renewable Feedstocks

**Figure 8:**
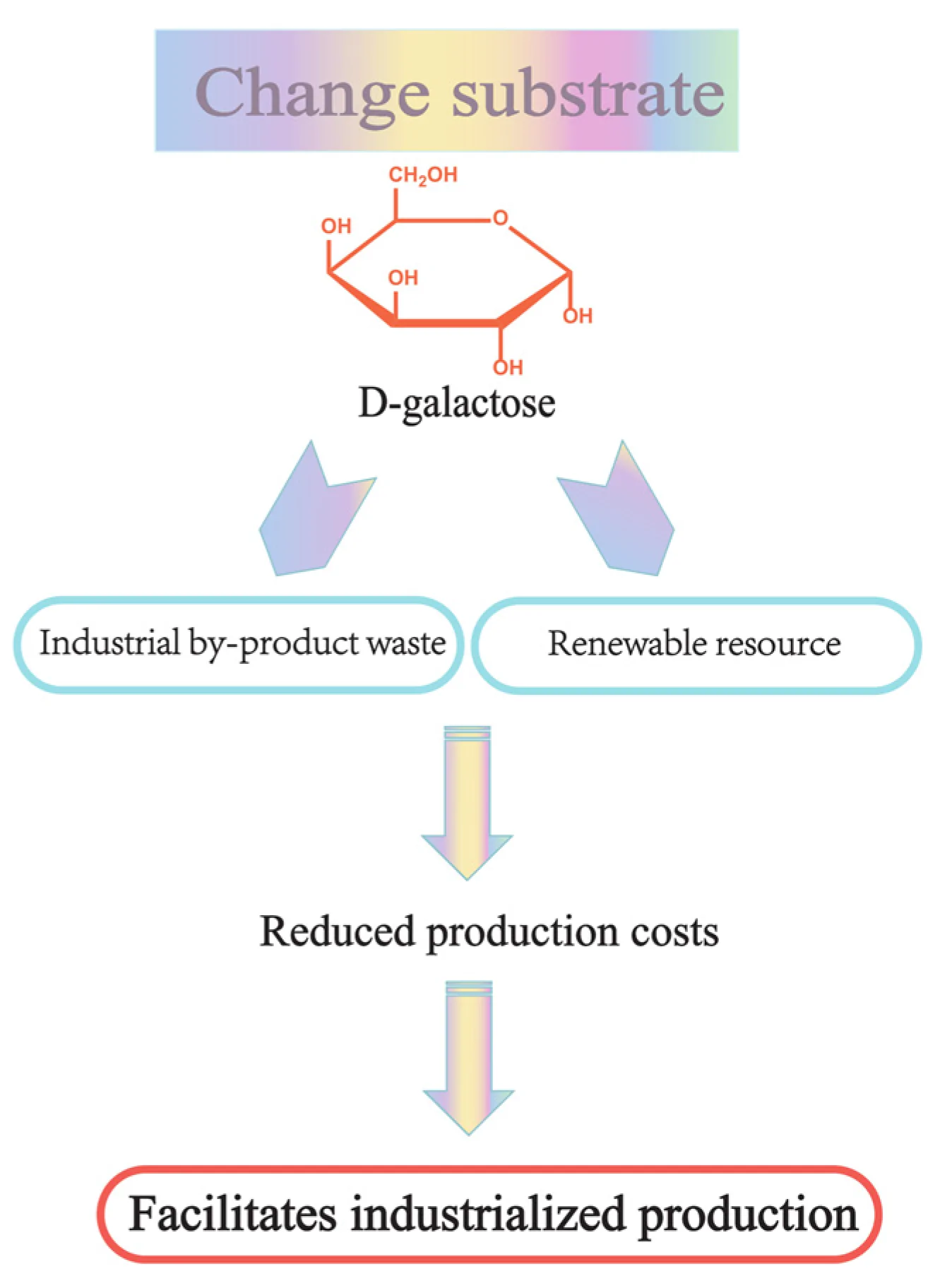
Changing substrates to facilitate industrial production.

Extensive L-AI characterization and protein engineering have improved enzyme properties and increased D-tagatose yields. However, these advances alone do not ensure the economic viability of large-scale L-AI production, as the cost of the substrate D-galactose is high and may not be suitable for industrial production due to economic considerations. Furthermore, researchers have recognized the potential of industrial biomass wastes for the production of high-value chemicals, and efforts to valorize these wastes are expanding [60,61]. A breakthrough in the industrial production of D-tagatose could be achieved if D-galactose is obtained from inexpensive industrial by-products or wastes such as lactose containing by-products, including whey and whey permeate (Figure 8). Accordingly, Zhang et al. [62] established an efficient method for the conversion of lactose to D-tagatose by co-expressing a fusion of L. lactis L-AI and S. thermophilus β-D-galactosidase in *E. coli*. The results showed that the conversion rate of D-tagatose was 42.4% after 15 h of reaction at 50 °C and pH 8.0 with 300 g/L lactose as the substrate. Meanwhile, Zhang et al. [63] also isomerized D-galactose to D-tagatose by co-expressing L-AI and β-D-galactosidase in *E. coli*, which eventually produced 68.35 g/L of D-tagatose. In addition, Zheng et al. [64] developed a one-step biotransformation method for the production of D-tagatose from lactose and cheese whey powder. They expressed L-AI from *B. coagulans* in *E. coli* and found that using 100 g/L lactose as the substrate at 50 °C for 12 h resulted in 23.5 g/L of tagatose. However, a key challenge in processing lactose containing industrial wastes is pH compatibility, as β-D-galactosidase hydrolysis of lactose in whey permeate is favored under acidic conditions, whereas most L-AIs are readily inactivated at low pH. One strategy to overcome this obstacle is to develop L-AIs that remain active under acidic conditions. Accordingly, Zhan et al. [65] obtained a mutant enzyme (Q299K) from *E. coli* by directed evolution that efficiently hydrolyzed more than 95% of the lactose and isomerized 43% of D-galactose to D-tagatose at 34 °C and pH 6.5. Similarly, Cervantes et al. [66] investigated a three-stage process for the production of D-tagatose from cheese whey. In order to increase the yield of D-tagatose, the study optimized key process conditions (pH, temperature, cofactors, etc.). The process comprised three steps: hydrolysis of lactose in whey concentrate using β-D-galactosidase from *B. longum*; removal of glucose using *Pichia pastoris*; and isomerization of D-galactose to D-tagatose using L-AI from *B. stearothermophilus*. This method produced a glucose free syrup containing a 1:2 (*w*/*w*) ratio of D-tagatose to D-galactose. In addition, Dai et al. [67] studied maltodextrin and found that the production of D-tagatose from maltodextrin using a multi-enzyme catalytic system showed limited productivity, which the authors attributed to an imbalance in the ratio of each enzyme and the flux of intermediates, so they increased the expression of the rate limiting enzyme and used modular and metabolic engineering to improve the productivity of D-tagatose. The results showed that the conversion rate of D-tagatose increased from 20.8% after 24 h to 25.2% after 3 h following optimization with 10 g/L maltodextrin as the substrate. Meanwhile, a synthetic biology strategy further expands dairy side stream valorization: engineered *E. coli* was configured to produce D-tagatose from lactose and whey permeate through the tagatose-6-phosphate pathway, demonstrating a growth-coupled route for using low-cost dairy feedstocks [68].

To identify additional low-cost feedstocks, researchers have investigated D-tagatose production from plant biomass. Kim et al. [69] successfully produced D-tagatose from onion juice residue. They used L-AI from *P. polymyxa* and hydrolyzed a 10% (w/v) onion juice residue suspension at 45 °C. After 24 h of reaction, they found that about 2.37 g of D-tagatose was produced with a galactose conversion of 90.4%. Marine biomass also offers substantial biodiversity and has become an important resource for drug discovery and other biotechnological applications [70]. Seaweed has been shown to be a potential biomass resource that can be used to produce important chemicals and bioethanol [71,72]. Recently, Jayamuthunagai et al. [73] investigated the galactose rich *Caulerpa racemosa* and found that it could be used for D-tagatose production. The authors extracted D-galactose from the seaweed and then enzymatically treated D-galactose with purified L-AI from *L. plantarum* MTCC1407, which yielded approximately 34.5% D-tagatose after 40 h of reaction at 50 °C and pH 7.0. Meanwhile, Zhang et al. [74] overexpressed L-AI from *L. plantarum* CY.6 in *E. coli* for D-tagatose production. In this process, the authors first hydrolyzed lactose in whey permeate to generate D-galactose and glucose, and then used whole cells containing L-AI to further bio-convert lactose to D-tagatose. The process achieved a D-galactose conversion efficiency of 36.8% and a D-tagatose yield of 51.5 g/L. *L. plantarum* CY.6 showed higher conversion capacity than previously reported *L. plantarum*. These studies suggest that the use of renewable resources or industrial by-product waste for D-tagatose production may facilitate its industrialization. A biorefinery study using *Gelidium sesquipedale* as a galactose source also demonstrated L-AI-mediated D-tagatose production [11]. More recent studies have broadened the feedstock portfolio. L-AI immobilized in a sodium alginate silica hydrogel was used to convert guar gum hydrolysate to D-tagatose, showing that galactomannan rich plant materials can serve as alternative galactose sources [75]. In parallel, a metabolically engineered *E. coli* process reversed the Leloir pathway to enable direct glucose-to-tagatose biosynthesis, reducing dependence on lactose-derived D-galactose [76].

### 4.3. Increasing Production Using Immobilized Biocatalysts

**Figure 9:**
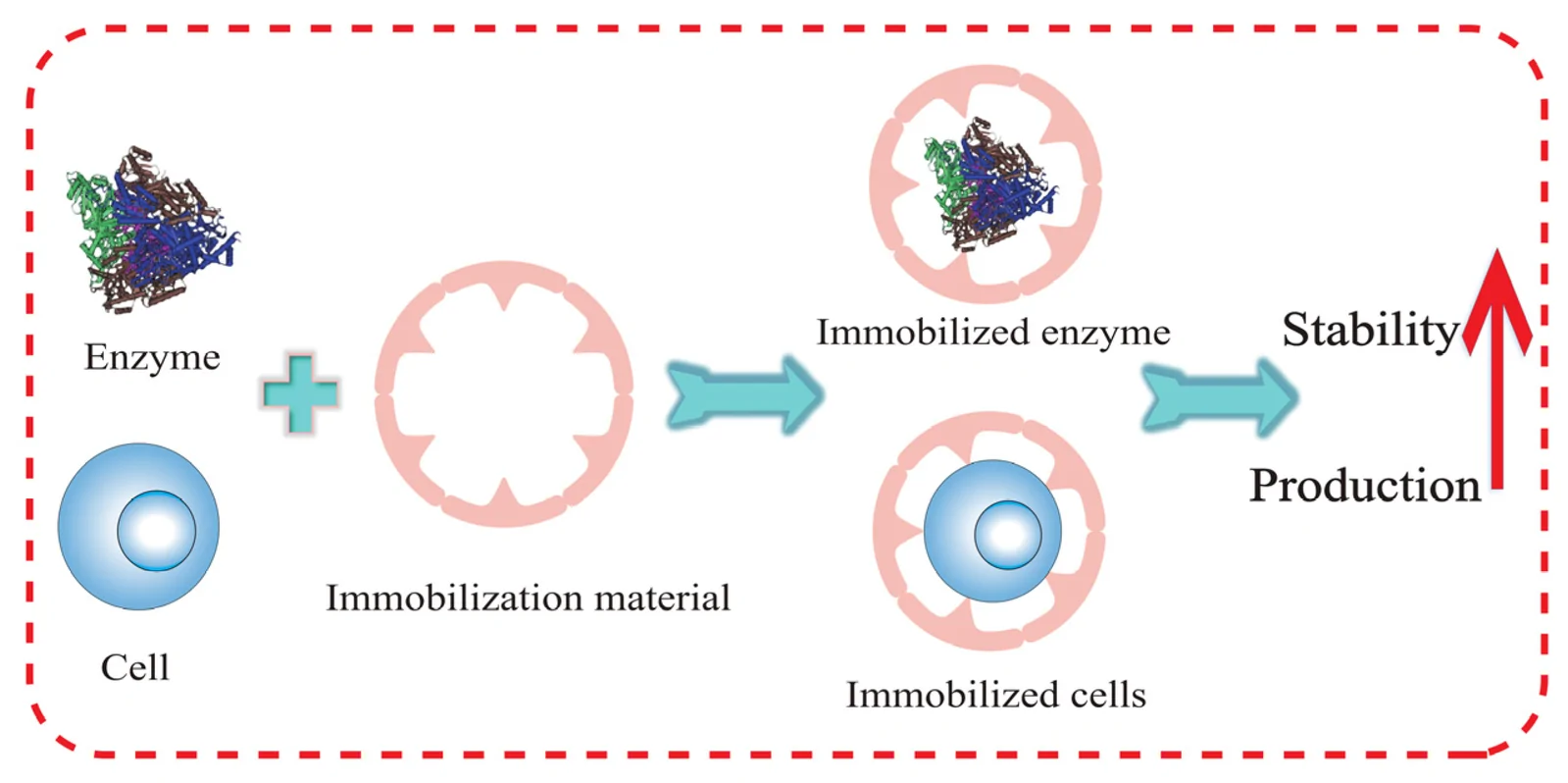
Immobilized enzymes and immobilized cells.

Given the high cost of enzymes, biocatalysts suitable for continuous or repeated use are preferred in industrial applications. However, since the isolation of these enzymes or whole cells containing biocatalysts from reaction systems is cumbersome and expensive, they are often not reused [77]. To address this problem, researchers have found that immobilization methods enable biocatalysts to be reused in a continuous process. It was found that several food grade enzymes (such as glucose isomerase and lactase) have been successfully applied in the industrial production of high fructose corn syrup and lactose by immobilization methods cross linked with gelatin or encapsulated into cellulose acetate fibers [78]. Studies indicate that immobilized cells or enzymes can increase D-tagatose productivity (Figure 9).

Immobilization technology has many advantages over free biocatalysts, such as (1) improved tolerance to harsh conditions; (2) increased D-tagatose titers; (3) enhanced operational stability over repeated cycles; (4) simplified downstream processing [79]. Currently, the use of immobilization technology for the production of xylitol or other sugars is widely reported, but immobilization technology for D-tagatose production still needs to be explored extensively [80,81]. In 2003, Ryu et al. [82] first evaluated D-tagatose production using L-AI encapsulated in sodium alginate. A packed bed bioreactor containing immobilized L-AI achieved a maximum average D-tagatose productivity of 1296 g/L/d under optimal conditions. Also, the authors found that the immobilized enzyme produced more than 50 g/L/h of D-tagatose continuously during the first 15 days, demonstrating the value of immobilization. In addition, they found a fourfold increase in the yield of the immobilized enzyme compared with previously reported values, with stability up to 40 days. Later, the group demonstrated the feasibility of using immobilized cells to produce D-tagatose. In one study, they compared the use of equal masses of immobilized cells and purified immobilized enzyme to produce D-tagatose and found that higher yields of D-tagatose could be obtained with immobilized cells than with immobilized enzyme, and they suggested that the reason for the low yield of immobilized enzyme could be the partial loss of enzyme during the purification step.

With the development of immobilization technology, Bortone et al. [83] immobilized *T. maritima* derived L-AI on a copper-chelated epoxy carrier with a 66% conversion of D-galactose at 80 °C. Rai et al. [84] immobilized L-AI of *L. sakei* in alginate beads and packed them into a reactor, and the results showed that the conversion of D-galactose was 50% after 24 h of reaction at 50 °C and pH 6.0. Salonen et al. [85] achieved overexpression of *B. longum* derived L-AI in *Lactococcus lactis* with a D-tagatose productivity of 7.708 g/L/h over 10 days and no significant decrease in conversion rate over ten bioconversion batches. Bober et al. [45] expressed L-AI of *L. sakei* origin in *L. plantarum* and treated the cells with the permeabilizing agent sodium dodecyl sulfate for 1 h. Using 300 mM D-galactose as the substrate, the D-galactose conversion rate reached 85% after 48 h at 50 °C. The authors attributed this to the selective permeability of the cell membrane of *L. plantarum*, which created concentration gradients of D-galactose and D-tagatose across the cell membrane, thus increasing the conversion rate of D-galactose. In addition, sodium dodecyl sulfate treatment removed cell-surface proteins and enhanced cell permeability, thereby increasing the conversion rate and providing a potential strategy for increasing D-tagatose yield. Recent immobilization studies have advanced both whole-cell and enzyme-based recycling. Li et al. [86] immobilized *E. coli* expressing the L-AI F118M/F279I variant in sodium alginate and increased D-tagatose production to 129.43 g/L at high substrate loading while maintaining substantial activity over repeated batches. In another study, immobilization of a newly characterized arabinose isomerase broadened the optimal temperature range and extended enzyme half-life, further supporting immobilization as a strategy for operationally stable L-AI biocatalysts [87].

## 5. Conclusions and Outlook

For decades, microorganisms and their enzymes have been a major source of biocatalysts and have been used for the production of valuable rare sugars [88,89]. Thanks to advances in molecular biology and certain biochemical techniques, the production of rare sugars has advanced considerably, but certain rare sugars such as D-tagatose still face substantial barriers to large-scale production. Key challenges include enzyme stability and conversion efficiency under harsh conditions. By performing targeted or random mutagenesis of the enzyme, researchers can obtain improved variants with strong acid tolerance, greater thermostability, and higher product yields, which is of great significance for improving the yield of D-tagatose. Future biotechnology-based D-tagatose production should prioritize sustainable feedstocks, robust catalysts, and efficient downstream processing. Recombinant strains may use hosts with an established history of food use or recognized safety status; however, recombinant construction does not itself confer GRAS status, and each strain and process requires appropriate safety and regulatory evaluation. Industrial development should consider titer, productivity, catalyst stability, purification cost, and overall process economics.

## Figures and Tables

**Table 1:** Properties of L-AI from different microbial sources.

Microorganism Source	Optima Temperature (°C)	Optimal pH	Metal Ion Activator	References
*L. fermentum* CGMCC2921	65	6.5	Mn^2+^, Co^2+^	[31]
*L. plantarum* WU14	60	7.17	Mn^2+^	[32]
*B. stearothermophilus* US100	80	7.5	Mn^2+^, Co^2+^	[33]
*B. stearothermophilus* IAM11001	65	7.5	Mn^2+^	[34]
*A. flavithermus*	95	10.5	Ni^2+^	[35]
*T. maritima*	90	7.5	Mn^2+^, Co^2+^	[36]
*B. thermoglucosidasius*	40	7.0	Mn^2+^	[37]
*T. neapolitana*	85	7.0	Mn^2+^, Co^2+^	[38]
*B. coagulans*	70	7.0	Mn^2+^	[39]
*L. parabuchneri* CICC 6004	45	6.7	Ca^2+^	[40]
*S. flexneri*	40	8.0	Mn^2+^, Co^2+^	[41]
*L. sakei* 23K	30–40	5.0–7.0	Mn^2+^, Mg^2+^	[42]
*B. coagulans* NL01	60	7.5	Mn^2+^, Co^2+^	[43]

## Data Availability

No new data were created or analyzed in this study. Data sharing is not applicable to this article.
